# Risk Factor Analysis of Acute Kidney Injury After Microwave Ablation of Hepatocellular Carcinoma: A Retrospective Study

**DOI:** 10.3389/fonc.2020.01408

**Published:** 2020-09-04

**Authors:** Yongfeng Yang, Fangyi Liu, Jie Yu, Zhigang Cheng, Zhiyu Han, Jianping Dou, Jie Hu, Ze Wang, Haigang Gao, Qiao Yang, Jing Tian, Yongjie Xu, Xiaoli Bai, Liping Lu, Ping Liang

**Affiliations:** ^1^Department of Interventional Ultrasound, Chinese PLA General Hospital, Beijing, China; ^2^Special Clinic Department, The 985th Hospital of Chinese People's Liberation Army Joint Logistic Support Force, Taiyuan, China; ^3^Ultrasonic Diagnosis Department, The 991st Hospital of Chinese People's Liberation Army Joint Logistic Support Force, Xiangyang, China; ^4^Ultrasound Department, Zunhua People's Hospital, Zunhua, China; ^5^Tianjin Institute of Urology, The Second Hospital of Tianjin Medical University, Tianjin, China; ^6^Department of Diagnostic Ultrasound, PLA Strategic Support Force Characteristic Medical Center, Beijing, China; ^7^Ultrasound Department, Hohhot Mongolian Hospital of Traditional Chinese Medicine, Hohhot, China; ^8^Department of Ultrasonography, Nanhai Hospital of Southern Medical University, Foshan, China

**Keywords:** acute kidney injury, microwave ablation, hepatocellular carcinoma, complication, risk factor analysis

## Abstract

**Objectives:** Acute kidney injury (AKI) is a recently observed side effect in patients after microwave ablation (MWA) of hepatocellular carcinoma (HCC) and is associated with negative outcomes. The aim of this study is to explore the risk factors of affecting the occurrence of AKI (stages 1b, 2, and 3), because they have a higher mortality rate than patients with AKI (stage 1a) and without AKI.

**Materials and methods:** In this retrospective study, a total of 1,214 patients with HCC who were treated with MWA under ultrasound (US) guidance in our department between January 2005 and November 2017 were enrolled. We evaluated the influence of 20 risk factors. Univariate and multivariate analysis were used for statistical analysis. The possible risk factors of AKI after MWA for HCC were summarized.

**Results:** AKI, AKI (stage 1a), and AKI (stages 1b, 2, and 3) after MWA were found in 34, 15, and 19 patients (2.80, 1.24, and 1.57%), respectively. Among 34 patients with AKI, 10 cases with AKI (stage 1a) and 6 cases with AKI (stages 1b, 2, and 3) recovered before their discharge without any treatment for AKI and 9 cases with AKI (stages 1b, 2, and 3) with further treatment. Four cases who had chronic renal failure before MWA of liver accepted renal dialysis. By univariate analysis, the number of antenna insertions (*P* = 0.027, OR = 3.3), MWA time ≥20 min (*P* = 0.029, OR = 4.3), creatinine (Cr)-pre above the upper limit of the reference value (*P* < 0.001, OR = 35.5), albumin (Alb)-pre (*P* = 0.030, OR = 0.9), and red blood cell (RBC)-pre (*P* < 0.001, OR = 0.3) were significant risk factors. By multivariate analysis, Cr-pre ≥ 110 μmol/L (*P* < 0.001, OR = 31.4) and MWA time ≥20 min (*P* = 0.043 OR = 9.9) were the independent risk factors.

**Conclusion:** AKI (stages 1b, 2, and 3) is a relatively serious complication after MWA for HCC, which is related to MWA time and Cr-pre. It requires attention by clinicians. So it is of great necessity to assess the Cr-pre level and reduce the MWA time to <20 min to minimize the risk of AKI after MWA for HCC.

## Introduction

Microwave ablation (MWA) is an important therapy for the focal HCC with single or up to three nodules (<3 cm), which is recommended by the 2018 Barcelona Clinic Liver Cancer (BCLC) system (1). As a minimally invasive therapy, MWA is safe with a low incidence of major complications, which was 0–2.7% (2–4), including 1.7% pleural effusion requiring thoracentesis, 1.4% tumor seeding, 0.4% liver abscess and empyema, 0.1% hemorrhage requiring arterial embolization, and 0.1% bile duct injury (5). Acute kidney injury (AKI) after MWA of HCC has been a recently observed complication, which is diagnosed by the following criteria—Stage 1: Creatinine ≥1.5 times baseline or increase of ≥0.3 mg/dl within any 48-h period (1a: Creatinine <1.5 mg/dl, 1b: Creatinine ≥ 1.5 mg/dl); Stage 2: Creatinine ≥ 2.0 times baseline; Stage 3: Creatinine ≥ 3.0 times baseline or increase to ≥4.0 mg/dl or acute dialysis (6–9). Ding et al. reported that the accidence of AKI is 23.6% for the large liver tumor (>5 cm) after MWA (10). Most importantly, the patients with AKI and creatinine >1.5 mg/dl (stages 1b, 2, and 3) present a worse clinical outcome. They had a higher mortality rate than patients with AKI stage 1a (Cr < 1.5 mg/dl) and without AKI. This fact reinforces that small elevations in the value of creatinine, especially when they exceed 1.5 mg/dl, have a great impact on the morbidity and mortality of patients with cirrhosis (6, 11). Furthermore, despite most of them having recovered, a few cases after RFA of metastasis liver cancer developed the renal failure, requiring intensive care unit admission and a prolonged hospital stay (12). Ong et al. summarized that the accidence of renal failure after MWA of liver tumors was 1.7% (13). Although there are reports in the existing literature regarding AKI after MWA of liver tumors including HCC, metastasis, and hemangioma, there are seldom exclusive reports on AKI for HCC in particular. Therefore, we determined the risk factors of AKI after MWA of HCC to prevent the occurrence of severe complications.

### Patients

The clinical data of 1,214 adult patients admitted for HCC with histopathological diagnosis and treated with ultrasound-guided percutaneous MWA from January 2005 to November 2017 were reviewed in this study.

### Evaluation Methods

Blood tests including routine, biochemistry, and coagulation function tests were conducted before and after MWA. In 1,214 patients, hepatic and renal functions were tested on the first day after MWA. Data of the MWA maximum diameter, the MWA parameter, and all blood test results of patients in this study were obtained from our departmental database. MWA energy was calculated by the equation E = P ^*^ T, where P and T were ablation power and time, respectively. The MWA zone was spherical in shape. The maximum diameter in the three dimensions of the tumor was measured using ultrasound. The comorbidity score was the pre-operation assessment of other diseases except for HCC such as diabetes, cardiovascular diseases, or AIDS using the Charlson comorbidity index (CCI) that included 19 diseases as well as the age of the patient (14). According to the anatomical segment of the liver, the location of the tumor was separated into four sections including the left lateral lobe, the left inner lobe (including the caudate lobe), the right anterior segment, and the right posterior segment. Because there were just nine patients whose tumor located in the caudate lobe, we attributed the caudate lobe into the left inner lobe to narrow deviation. The number of antenna insertions was defined as the total number of antenna placements in each patient during ablation.

### MWA Equipment and Technology

The MWA unit used was a 100 W two-cooled-shaft system (KY-2000, Kangyou Medical, Nanjing, China) with frequencies of 2,450 and 915 MHz. The antennae (KY-2450-T11b, KY-2450B-T3, 89 KY-2450B-T7 and KY-2450B-QT) were percutaneously inserted into the tumor and placed at a designated location under ultrasound guidance. When the distance of tumor to the important structure such as the main bile, gallbladder, and bowel was <5 mm, the thermocouple needles can be inserted at the margin of those structure to monitor temperature in real time. To reduce the risk of the bleeding and the seeding of tumor, the MW emission was continued until the antennae were withdrawn to below the skin entrance site after the MWA of the tumor (4).

### Statistical Analysis

Data analysis was performed using EmpowerStats (Version 3.4.3) for Windows, and the continuous data were expressed as β (95%CI) *P*-value/OR (95%CI) *P*-value. All of the analyses were performed with statistical software package R (http://R-project.org, The R-foundation) and EmpowerStats (http://empowerstats.com, X&Y Solutions. Inc., Boston, MA). Data of two groups were analyzed between the group A (no AKI and AKI 1a) and group B (AKI stage 1b,2,3) by using the Student *t* test for unpaired data and Fisher exact test as appropriate. Twenty related risk factors, including gender, age, comorbidity scores, biochemical parameters, and blood routine before treatment like alanine transaminase (ALT)-pre, glutamic oxaloacetic transaminase (AST)-pre, ALB-pre, STB-pre, Cr-pre, Hb-pre, platelet (PLT)-pre, white blood cell (WBC)-pre, lymphocyte (LY)-pre, red blood cell (RBC)-pre, the location of tumor, the maximum diameter of tumor, MWA energy, MWA time, and the number of antenna insertions, were analyzed using the univariate and multivariate logistic regression model method.

## Results

### Basic Analysis

Among all 1,214 patients after MWA of HCC, 19 patients had AKI (stages 1b, 2, and 3), and the accidence was 1.57%. The biochemical parameters and blood routine before treatment of all patients, tumor characteristic, and MWA parameters were described. The mean maximum diameter of tumor for 829 patients with solitary tumor with AKI vs. without AKI was 3.0 vs. 3.8 cm, respectively. The mean ALT, AST, and STB for the 19 patients were 32.8 U/L, 31.1 U/L, and 13.9 μmol/L, respectively. The mean ALT, AST, and STB for 1,195 patients without AKI were 32.7 U/L, 33.7 U/L, and 16.8 μmol/L. Most *P*-values were >0.05 except Hb-pre, RBC-pre, and Alb-pre. There were no significant differences between the groups. The basic characteristic is shown in Table 1.

**Table 1:** Baseline characteristic of acute kidney injury (AKI) (stages 1b, 2, and 3).

**Variation**	**Total**	**No AKI and AKI 1A**	**AKI 1B,2, and 3**	***P*-value**
Patients no.	1,214	1,195	19	
Age(year)	58.4 ± 10.8	58.4 ± 10.8	56.8 ± 11.4	0.535
Gender				0.798
Female	228 (18.8%)	224 (18.7%)	4 (21.1%)	
Male	986 (81.2%)	971 (81.3%)	15 (78.9%)	
ALT-pre(U/L)	32.7 ± 24.5	32.7 ± 24.4	32.8 ± 29.6	0.977
AST-pre(U/L)	33.7 ± 25.6	33.7 ± 25.8	31.1 ± 14.4	0.67
ALB-pre(g/L)	39.5 ± 5.0	39.5 ± 5.0	37.1 ± 5.6	**0.036**
STB-pre(mol/L)	16.8 ± 9.4	16.8 ± 9.4	13.9 ± 7.3	0.176
Cr-pre(μmol/L)	77.1 ± 58.0	74.1 ± 37.1	261.5 ± 314.2	** <0.001**
				** <0.001**
<110	1,182 (97.4%)	1,171 (98.0%)	11 (57.9%)	
≧110	32 (2.6%)	24 (2.0%)	8 (42.1%)	
HB-pre(g/L)	135.1 ± 18.7	135.4 ± 18.4	118.2 ± 23.9	** <0.001**
PLT-pre(10^9^/L)	119.6 ± 59.3	119.6 ± 59.5	120.1 ± 48.6	0.973
LY-pre(10^9^/L)	0.4 ± 2.2	0.4 ± 2.2	0.3 ± 0.1	0.829
WBC-pre(10^9^/L)	4.7 ± 4.7	4.7 ± 4.8	4.6 ± 2.0	0.925
NE-pre(10^9^/L)	0.6 ± 0.1	0.6 ± 0.1	0.6 ± 0.1	0.096
RBC-pre(10^13^/L)	4.3 ± 0.6	4.3 ± 0.6	3.8 ± 0.9	** <0.001**
MWA time(s)	723.2 ± 457.4	721.6 ± 457.9	825.8 ± 423.1	0.325
				0.164
<600	568 (46.8%)	563 (47.1%)	5 (26.3%)	
≧600, <900	322 (26.5%)	315 (26.4%)	7 (36.8%)	
≧900, <1,200	163 (13.4%)	161 (13.5%)	2 (10.5%)	
≧1,200	161 (13.3%)	156 (13.1%)	5 (26.3%)	
Comorbidity scores				0.597
0	172 (14.2%)	169 (14.2%)	3 (16.7%)	
1	245 (20.2%)	243 (20.4%)	2 (11.1%)	
2	276 (22.8%)	273 (22.9%)	3 (16.7%)	
3	224 (18.5%)	221 (18.5%)	3 (16.7%)	
≧4	294 (24.3%)	287 (24.1%)	7 (38.9%)	
The location of tumor				0.476
The left lateral lobe	147 (12.1%)	145 (12.1%)	2 (10.5%)	
Left inner lobe(including caudate lobe)	158 (13.0%)	157 (13.1%)	1 (5.3%)	
Right anterior segment	445 (36.7%)	435 (36.4%)	10 (52.6%)	
Right posterior segment	464 (38.2%)	458 (38.3%)	6 (31.6%)	
Maximum diameter of tumor (cm)	3.0 ± 1.5	3.0 ± 1.5	3.5 ± 1.9	0.100
				0.497
≦3	776 (63.9%)	766 (64.1%)	10 (52.6%)	
>3, ≦5	324 (26.7%)	318 (26.6%)	6 (31.6%)	
>5	114 (9.4%)	111 (9.3%)	3 (15.8%)	
Tumor no.				0.994
Solitary	829 (68.3%)	816 (68.3%)	13 (68.4%)	
Multiple	384 (31.7%)	378 (31.7%)	6 (31.6%)	
MWA energy(J)	38,473.7 ± 26,042.4	38,373.5 ± 26,052.7	44,763.2 ± 25,258.4	0.289
The number of electrodes				0.354
1	274 (22.6%)	272 (22.8%)	2 (10.5%)	
2	840 (69.2%)	824 (69.0%)	16 (84.2%)	
≧3	100 (8.2%)	99 (8.3%)	1 (5.3%)	
The number of antenna insertions				0.078
1–2	634 (52.2%)	628 (52.6%)	6 (31.6%)	
3–4	359 (29.6%)	349 (29.2%)	10 (52.6%)	
≧5	221 (18.2%)	218 (18.2%)	3 (15.8%)	

*Results in table, Mean + SD/N (%). the Cr-pre, 1 mg/dl = 88.4 μmol/L*.

*Bold values represent P < 0.05*.

### Risk Factors of AKI

By univariate analysis, the number of antenna insertions (*P* = 0.027, OR = 3.3), MWA time ≥ 20 min (*P* = 0.029, OR = 4.3), Cr-pre above the upper limit of the reference value (*P* < 0.001, OR = 35.5), Alb-pre (*P* = 0.030, OR = 0.9), and RBC-pre (*P* < 0.001, OR = 0.3) were significant risk factors. The maximum diameter of tumor for patients with solitary tumor was analyzed by univariate analysis separately (Table 2). While by univariate and multivariate analysis, Cr-pre ≥ 110 μmol/L (*P* < 0.001, OR = 31.4) and MWA time ≥20 min (*P* = 0.043 OR = 9.9) were the independent risk factors associated with AKI (stages 1b, 2, and 3) (Tables 3, 4).

**Table 2:** Baseline and univariate analysis of maximum diameter for patients with solitary tumor.

**Variation**	**Total**	**No AKI and AKI 1A**	**AKI(stages 1B, 2, and 3)**	***P*-value**	**OR (95%CI) *P*-value**
Tumor No.	829	816	13		
Maximum diameter of tumor (cm)	3.0 ± 1.6	3.0 ± 1.5	3. ± 2.1	0.047	
				0.114	
≦3	529 (63.8%)	524 (64.2%)	5 (38.5%)		1
>3, ≦5	217 (26.2%)	212 (26.0%)	5 (38.5%)		2.5 (0.7, 8.6) 0.156
>5	83 (10.0%)	80 (9.8%)	3 (23.1%)		3.9 (0.9, 16.8) 0.064

**Table 3:** Univariate analysis for acute kidney injury (AKI) (stages 1b, 2, and 3) for risk factors.

**Exposure**	**Solitary**	**Total**
**Gender**		
Female	1	1
Male	0.9 (0.2, 3.2) 0.817	0.9 (0.3, 2.6) 0.800
Age(year)	1.0 (0.9, 1.0) 0.507	1.0 (0.9, 1.0) 0.531
**Comorbidity scores**		
0	1	1
1	0.5 (0.1, 2.9) 0.418	0.5 (0.1, 2.8) 0.401
2	0.2 (0.0, 2.0) 0.177	0.6 (0.1, 3.1) 0.558
3	0.5 (0.1, 3.2) 0.481	0.8 (0.2, 3.8) 0.742
≧4	0.8 (0.2, 3.5) 0.725	1.4 (0.4, 5.4) 0.647
**The location of tumor**		
The left lateral lobe	1	1
Left inner lobe(including caudate lobe)	1.0 (0.1, 16.5) 0.989	0.5 (0.0, 5.1) 0.528
Right anterior segment	2.3 (0.3, 18.9) 0.438	1.7 (0.4, 7.7) 0.510
Right posterior segment	1.4 (0.2, 12.8) 0.757	0.9 (0.2, 4.7) 0.946
**Maximum diameter of tumor (cm)**		
≦3	1	1
>3, ≦5	2.5 (0.7, 8.6) 0.156	1.4 (0.5, 4.0) 0.476
>5	3.9 (0.9, 16.8) 0.064	2.1 (0.6, 7.6) 0.275
ALT-pre(U/L)	1.0 (1.0, 1.0) 0.715	1.0 (1.0, 1.0) 0.982
AST-pre(U/L)	1.0 (1.0, 1.0) 0.873	1.0 (1.0, 1.0) 0.660
ALB-pre(g/L)	0.9 (0.8, 1.0) 0.083	**0.9 (0.8, 1.0) 0.030**
STB-pre(mol/L)	1.0 (0.9, 1.1) 0.776	1.0 (0.9, 1.0) 0.171
Cr-pre(μmol/L)	1.0 (1.0, 1.0) <0.001	1.0 (1.0, 1.0) <0.001
<110	1	1
≧110	**19.7 (5.5, 70.0)** **<** **0.001**	**35.5 (13.1, 96.0)** **<** **0.001**
HB-pre(g/L)	1.0 (0.9, 1.0) 0.027	1.0 (0.9, 1.0) <0.001
PLT-pre(10^9^/L)	1.0 (1.0, 1.0) 0.993	1.0 (1.0, 1.0) 0.965
LY-pre(10^9/^L)	0.1 (0.0, 35.9) 0.428	0.0 (0.0, 0.6) 0.029
WBC-pre(10^9^/L)	0.9 (0.6, 1.3) 0.674	1.0 (0.9, 1.1) 0.929
NE-pre(10^9^/L)	1.4 (0.0, 286.8) 0.912	41.4 (0.5, 3,189.7) 0.093
RBC-pre(10^13^/L)	0.4 (0.2, 1.1) 0.069	**0.3 (0.1, 0.6)** **<** **0.001**
The number of electrodes		
1	1	1
2	1.5 (0.3, 7.0) 0.594	2.6 (0.6, 11.6) 0.197
≧3	0.9 (0.1, 9.5) 0.897	1.4 (0.1, 15.6) 0.803
**MWA time(s)**		
<600	1	1
≧600, <900	1.2 (0.2, 6.4) 0.806	2.9 (0.9, 9.6) 0.082
≧900, <1,200	2.4 (0.5, 12.8) 0.290	1.6 (0.3, 8.7) 0.574
≧1,200	5.7 (1.5, 21.8) 0.011	**4.3 (1.2, 15.8) 0.029**
**The number of antenna insertions**		
1–2	1	1
3–4	**4.1 (1.3, 13.0) 0.018**	**3.3 (1.1, 9.6) 0.027**
≧5	1.5 (0.2, 12.9) 0.717	1.7 (0.4, 7.6) 0.478
MWA energy(J)	1.0 (1.0, 1.0) 0.047	1.0 (1.0, 1.0) 0.266

*Results in table, β (95%CI) P value/OR (95%CI) P value. Bold values represent P < 0.05*.

**Table 4:** Multivariate analysis of acute kidney injury (AKI) (stages 1b, 2, and 3) for risk factors.

**Exposure**	**Solitary**	**Total**
ALB-pre(g/L)	0.9 (0.8, 1.0) 0.081	0.9 (0.8, 1.0) 0.091
STB-pre(mol/L)	1.0 (0.9, 1.0) 0.436	1.0 (0.9, 1.0) 0.186
**Cr-pre(μmol/L)**		
<110	1	1
≧110	41.0 (6.9, 243.4) <0.001	**31.4 (8.2, 120.2)** **<** **0.001**
HB-pre(g/L)	1.0 (0.9, 1.0) 0.245	1.0 (0.9, 1.0) 0.600
NE-pre(10^9^/L)	0.1 (0.0, 40.8) 0.435	10.5 (0.1, 1,712.3) 0.366
RBC-pre(10^13^/L)	3.5 (0.6, 19.4) 0.150	1.3 (0.3, 5.8) 0.713
**MWA time(s)**		
<600	1	1
≧600, <900	0.6 (0.0, 7.3) 0.656	1.7 (0.3, 10.2) 0.564
≧900, <1,200	1.0 (0.1, 16.4) 0.990	0.9 (0.1, 10.2) 0.924
≧1,200	11.5 (0.7, 180.5) 0.081	**9.9 (1.1, 91.3) 0.043**
**The number of antenna insertions**		
1–2	1	1
3–4	4.2 (0.4, 45.7) 0.233	3.0 (0.5, 16.9) 0.222
≧5	0.3 (0.0, 8.6) 0.508	0.4 (0.0, 4.2) 0.406
**Maximum diameter of tumor (cm)**		
≦3	1	1
>3, ≤ 5	1.7 (0.4, 8.4) 0.503	1.4 (0.4, 4.8) 0.636
>5	1.7 (0.2, 14.1) 0.636	0.9 (0.2, 4.8) 0.857

*Results in table, β (95%CI) P value/OR (95%CI) P value. Bold values represent P < 0.05*.

## Discussion

AKI (stage 1a) is a transient and controlled complication for most patients after MWA of HCC, but AKI (stages 1b, 2, and 3) needs further treatment. According to our research, among 34 patients with AKI after MWA, 10 cases with AKI (stage 1a) and 6 cases with AKI (stages 1b, 2, and 3) recovered before their discharge without any treatment for AKI whose mean post-operation Cr level was 123.2 μmol/L. Five cases with AKI (stage 1a) and nine cases with AKI (stages 1b, 2, and 3) recovered after further treatment of renal conservation: (1) the diuretic-furosemide or/and spironolactone and (2) sodium bicarbonate injection. Their mean post-operation Cr was 169.8 μmol/L. Then, four cases accepted the renal dialysis who had the chronic renal failure before MWA of liver whose mean post-operation Cr level was 947.4 μmol/L. Hence, most cases of AKI after MWA for HCC had a good recovery unless with chronic kidney disease (CKD), but the hospital stay was prolonged (10). What is worse, the patients with AKI (stages 1b, 2, and 3) had a higher mortality rate than patients with AKI stage 1a (Cr < 1.5 mg/dl) and without AKI. Lins et al. reported that the mortalities of the cirrhotic patient group with (A) no AKI, (B) AKI (stage 1a), (C) AKI (stage 1b), and (D) AKI (stages 2 and 3) were 11.8, 12.5, 33.3, and 52.4%, respectively (6). Fagundes et al. presented that the survival rates of groups B, C, and D were 84, 68, and 36%, respectively (*p* < 0.001) (11). Furthermore, Rodriguez et al. reported that three patients after radiofrequency ablation (RFA) of metastasis liver cancer developed renal failure, requiring intensive care unit admission and a prolonged hospital stay (12), even though there was no report about acute renal failure after MWA of HCC without preexisting CKD. It is still of great necessity to pay much attention to the risk factor of AKI (stages 1b, 2, and 3) after MWA to prevent the severe complication happening and improve the survival.

This study shows that the statistically significant risk factors for AKI (stages 1b, 2, and 3) after MWA for HCC were 3 ≤ the number of antenna insertions ≤ 4 (*P* = 0.027, OR = 3.3) RBC-pre (*P* < 0.001, OR = 0.3). For the patients with multiple tumors, Hb-pre (*P* < 0.001, OR = 0.9) were also significant risk factors. MWA time ≥ 20 min (*P* = 0.029, OR = 4.3) was the independent risk factor by multivariate analysis. As we know, the thermal effect of ablation can lead to the destruction of RBC and the release of Hb to the circulation system. When the quantity of cell-free Hb exceeds the liver detoxification threshold, some Hb will cross the glomerular filtration membrane, reach the renal tubules, and cause renal tubular necrosis that ultimately leads to AKI (15–18). The ablation of large tumors took longer time and more antenna insertions, and more Hb was released into the circulation system. Therefore, patients who had a high HB-pre level and RBC-pre and took longer time were more prone to having AKI than those who have a small tumor. Hence, the number of antenna insertions should be reduced for treating large and multiple tumors. It is recommended that the MWA time is controlled within 20 min.

Additionally, another independent risk factor was Cr-pre above the upper limit of the reference value (*P* < 0.001, OR = 31.4). In our analysis, there were six patients with AKI after MWA for HCC with CKD, and the preoperative Cr in these patients were higher than the upper level of the reference range (110 μmol/l). Multivariate analysis showed that Cr-pre > 110 μmol/L was an independent risk factor. The present literature showed that the mortality of people who had CKD with AKI was higher than those without AKI (19, 20), especially for critically ill patients with CKD (21). Therefore, when Cr-pre is above the upper limit of the reference value, the clinician should assess the patient's magnitude of benefit from MWA and then decide whether to do it. It was noted that the Alb-pre (*P* = 0.030, OR = 0.9) was also a significant risk factor. It was revealed that the infection before MWA could make it easier to AKI for patients. Then, the low levels of STB-pre and Alb-pre were also significant risk factors. The mechanism was still unclear.

Therefore, to avoid complications of AKI (stages 1b, 2, and 3), the independent risk factors reported in this study should be emphasized in the preoperative evaluation. Clinicians should pay attention to the Cr-pre and the need for long MWA time. Appropriate measures should be taken including preoperative evaluation of renal function, intraoperative and postoperative administration of fluid, appropriate timing of diuresis, and urine alkalization (10, 17, 22). Furthermore, patient survival should actively involve not only the preservation of their kidney health but also postdischarge follow-up of kidney function because severe AKI predisposes patients to faster progression of CKD later on—especially when they had multiple hits of AKI or preexisted CKD (23).

The innovation of the study is that the risk factor of the maximum diameter of tumor in patients with single nodule was analyzed separately, and the interference of cases with multiple tumors on this factor was excluded. It is because the maximum diameter is just referred to the largest one among multiple tumors for them. That cannot reflect the relation of the size of tumor to the AKI accurately.

There are three limitations of this research. First, this is a retrospective study. To provide further evidence for the conclusion, prospective studies are still needed. Then, the clinical data were just collected from a single institution. Finally, we designed this study to assess the Cr only on the first day after MWA in the hospital; we did not evaluate the occurrence of late AKI. So the occurrence incidence of the AKI after MWA of HCC we obtained may be lower than the true value.

## Data Availability Statement

All datasets generated for this study are included in the article/supplementary material.

## Ethics Statement

The studies involving human participants were reviewed and approved by Ethics Committee of PLA General Hospital. The patients/participants provided their written informed consent to participate in this study.

## Author Contributions

YY, FL, and PL made substantial contributions to the research design, analysis, and interpretation of data. FL contributed a lot to the data analysis and appropriate method to the research. ZC and ZH took part in the building of the clinical database. JY provided the instruction for statistical analysis by EmpowerStats (Version 2019-11-18). JD, JH, ZW, HG, QY, JT, YX, XB, and LL performed the clinical collection. YY and FL drafted the manuscript. All authors contributed to the article and approved the submitted version.

## Conflict of Interest

The authors declare that the research was conducted in the absence of any commercial or financial relationships that could be construed as a potential conflict of interest. The reviewer CA declared a past co-authorship with several of the authors ZH, JD, JY, PL, ZC, and FL to the handling Editor.
